# A fully automatic system for acid-base coulometric titrations

**DOI:** 10.1155/S1463924690000323

**Published:** 1990

**Authors:** A. Cladera, A. Caro, J. M. Estela, V. Cerdà

**Affiliations:** Department of Analytical Chemistry Faculty of Sciences University of the Balearic Islands Palma de Mallorca 07071 Spain

## Abstract

An automatic system for acid-base titrations by electrogeneration of H^+^ and OH^-^ ions, with potentiometric end-point detection, was developed. The system includes a PC-compatible computer for instrumental control, data acquisition and processing, which allows up to 13 samples to be analysed sequentially with no human intervention.

The system performance was tested on the titration of standard solutions, which it carried out with low errors and RSD. It was subsequently applied to the analysis of various samples of environmental and nutritional interest, specifically waters, soft drinks and wines.


Journal of Automatic Chemistry, Vol. 12, No. 6 (November–December 1990), pp. 258–262

### A fully automatic system for acid–base coulometric titrations

**A. Cladera, A. Caro, J. M. Estela and V. Cerdà**

*Department of Analytical Chemistry, Faculty of Sciences, University of the Balearic Islands, 07071 Palma de Mallorca, Spain*

*An automatic system for acid–base titrations by electrogeneration of $H^+$ and $OH^-$ ions, with potentiometric end-point detection, was developed. The system includes a PC-compatible computer for instrumental control, data acquisition and processing, which allows up to 13 samples to be analysed sequentially with no human intervention.*

*The system performance was tested on the titration of standard solutions, which it carried out with low errors and RSD. It was subsequently applied to the analysis of various samples of environmental and nutritional interest, specifically waters, soft drinks and wines.*

### Introduction

Acid–base determinations are often included in environmental and food quality control studies. Hence the interest in developing fast, straightforward analytical methods allowing large numbers of samples to be assayed at the lowest possible cost.

Coulometric titrations have been used successfully by various authors [1,2] for the analysis of different foods. Some authors have used computers to automate this type of analysis and have applied their set-ups to the determination of strong [3] and weak acids [4]. Advantages of coulometric titrations for routine analyses for acids and bases include not needing to prepare and store standards, and the favourable determination limits and precision afforded. In addition, the use of a coulometric autotitrator substantially facilitates analyses by reducing human intervention to a minimum.

The use of standard personal computers, data acquisition boards and other commercially available instruments has allowed the development of automatic systems that can be readily assembled in the laboratory without a particularly strong knowledge of electronics.

In a previous paper [5] we reported on the development of a coulometric autotitrator prototype for the determination of chloride in water with electrogeneration of $Ag^+$ ions. This paper describes its adaptation to acid–base titrations.

### Experimental

### *Reagents*

The solutions used included 2 M KCl, 0·8 M $Na_2SO_4$, 2 M $KNO_3$, 0·1 N NaOH (standardized with potassium bipthalate) and 0·1 N HCl (standardized with $Na_2CO_3$). All reagents used were Merck *pro analysi.*

### *Apparatus*

The automated coulometric titration system used consisted of the following elements:

(1) An IBM PS/30 computer (any other PC compatible would be satisfactory), provided with a standard RS-232C serial interface.

(2) A 14-bit, 16-channel FPC-011 analogue/digital converting board (conversion time less than 42 μs), and a FPC-046 industrial board provided with 16 independent relays, both from Flytech Technology Co., Ltd (Taiwan), both located in the expansion slots of the PC.

(3) A microSAMPLER 2040 autosampler and a microBUR 2031 autoburette, both of which were provided with an RS-232C interface and supplied by Crison Instruments S.A. (Riera Principal 24–26, 08328 Alella, Barcelona, Spain).

(4) An AMEL 555C (Milan, Italy) potentiostat/galvanostat.

(5) An Ingold (Switzerland) Pt-ring generating electrode and a Pt-gauze counter electrode.

(6) A HANNA Instruments (Italy) HI-1911B combined glass electrode, self-furnished with an impedance-control amplifier placed in the head of the electrode and powered with a mercury cell. The special performance of this new electrode allows it to be directly connected to the A/D converter.

The arrangement of the above elements in the instrumental apparatus is shown in figure 1. Switches $R_1$–$R_4$ are computer-controlled relays, which allow passage of the current through the cell to be controlled. To start an experiment, the computer closes the circuit at $R_1$ and $R_3$, with which the generating electrodes are connected to the galvanostat. To stop the current, relays $R_1$ and $R_3$ are switched off, and $R_2$ and $R_4$ are switched on. The electrodes being isolated from the galvanostat, the current then passes through a 332 ohm metallic film resistor (1%, 50 ppm/°C), previously calibrated (see later). In this condition, the computer can measure the potential drop across the resistance via the A/D board, which in turn allows it to determine the current intensity supplied by the galvanostat.

The titration curve is acquired through the A/D converting board, which is connected to the indicator electrode via an *RC* filter ($R$ = 213 kohm, $C$ = 6·8 μF) through a channel other than that used to measure the current.

As can be seen in figure 1, the Pt counter electrode is located in a compartment containing 2 M $KNO_3$ and is isolated from the titration cell. Electrical contact between

 0142–0453/90 $3.00 © 1990 Taylor & Francis Ltd.

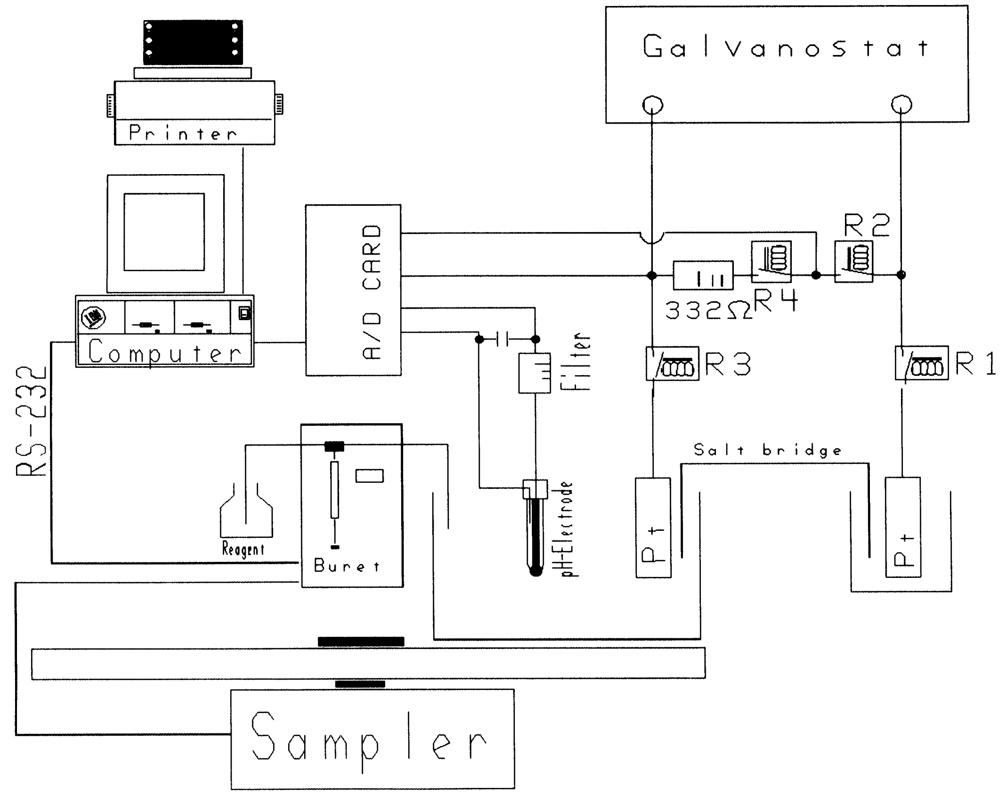

*Figure 1. Block diagram of the instrumental apparatus.*

the two compartments is accomplished through a salt bridge filled with $KNO_3$-saturated agar-agar.

The computer-controlled automatic sampler allows up to 13 samples to be assayed sequentially with no human intervention. The autoburette used, also controlled by the computer via an RS-232C interface, allows some reagent (or sample) to be added during the analyses.

*Software*

The instrumental set-up was controlled by the program COULOM, written by the authors in QuickBASIC†, which allows the acquisition and processing of the titration curves. Data are stored in ASCII and may be manipulated by other standard programs, for example LOTUS 1-2-3 and GRAPHER.

Basically, it consists of three distinct parts: calibration, acquisition of the titration curves, and data processing.

*Calibration*

The program includes two calibration subroutines. One of them allows the 332-ohm metallic film resistor (1%, 50 ppm/°C) to be calibrated in order to measure the current circulating through the system at a given time. Such a calibration is accomplished by supplying a known current intensity. This allows any intensity supplied by a constant-current source to be determined at a later stage by using the calibrated value of the resistance.

†*This program can be requested from SCIWARE at the Association of Environmental Sciences and Techniques, Department of Chemistry, Faculty of Sciences, Universitat de les Illes Balears, 07071 Palma de Mallorca, Spain.*

The second subroutine allows results to be expressed in pH units by calibration with the combined glass electrode. Calibrations are carried out with buffers of pH 4 and 7 in much the same way as is done for pH-meters. This subroutine was not present in the old release of COULOM.

*Acquisition of titration curves*

The program allows three types of titration to be controlled: continuous addition, multiple fixed-time additions, and multiple variable-time additions.

In continuous titrations, once the current supplied by the galvanostat has been measured, it is passed through the titration cell as data acquisition is started, and is not interrupted until the end of the titration.

In titrations with fixed-time additions, the current is passed for a preset time, after which the indicator electrode is allowed to stabilize and a point of the titration curve is acquired. This process is repeated until the titration is finished.

Titrations with variable-time additions are similar to fixed-time addition except that the time over which the current is passed is not fixed, but, rather, is calculated by the program from the evolution of the titration curve (the time decreases as the curve approaches the equivalence point). This process is thus identical to manual titration procedures.

The continuous option is the most practical of all three because it provides results similar to those obtained with the other two, but in a shorter time.

The program also allows a given amount of the titrant to be electrogenerated prior to starting data acquisition by any of the three options. This is a new feature of COULOM, which allows one to reduce the amount of memory used for the acquisition and storage of data from highly concentrated samples. This initial addition can be preset by the user or calculated by the computer from the potential across the titration cell and the expected equivalence potential.

The program allows all the samples held in the sampler to be assayed successively and independently, i.e. it allows the working parameters (titration method, final titration potential, data acquisition rate, etc.) to be set for each. It analyses each of the samples according to the specified parameters and rinses the electrodes between samples. Optionally, it saves the experimental data to disk and/or processes them to deliver a report of the results through the printer.

*Data processing*

Acquired data can be processed in two ways, namely by selecting a fixed potential, or pH, as the end-point, or by calculating these from the inflection point of the titration curve.

The first case is a new feature of COULOM, which is useful in a number of applications, for example in acidity determination of wines.

In the other case, the program determines the inflection point of the titration curve by calculating the first and second derivative of the experimental curve using the algorithm proposed by Savitzky–Golay [6], using the corrected tables of Steiner *et al.* [7]; the equivalence point will be the maximum of the first derivative, which will be marked by a sign change in the second. The use of the Savitzky–Golay algorithm (quadratic order) allows the simultaneous smoothing of the titration curve. The selected window for smoothing was the number of points corresponding to 2 μeq (approximately 19 points). The data treatment subroutine of COULOM developed for chloride determination had to be changed in order to permit automatic treatment of both increasing and decreasing potentiometric curves.

*Procedures*

*Titration of acids with electrogenerated hydroxyl ions*

A known sample volume and 10 ml of 2 M KCl were placed in the sampler cups, which were made up to volume (40 ml) with distilled water. For convenience, the addition of distilled water to adjust the final volume was performed by the program through the autoburette prior to the titration of each sample. Then, the current intensity supplied by the galvanostat was set in such a way that the generating electrode acted as the cathode and the external auxiliary Pt electrode functioned as the anode. The program for automatic analysis of the samples was then started.

*Titration of bases with electrogenerated hydrogen ions*

The experimental procedure was identical to that used for acids. In order to avoid chlorine evolution, 0·8 M $Na_2SO_4$, rather than 2 M KCl, was used. The generating electrode acted as the anode.

Unless otherwise stated, the results reported here were obtained by using the continuous titration method at a current intensity of 10 mA. Both types of sample require the acidity or basicity of the electrolyte to be taken into account, i.e. blanks need to be used to make appropriate corrections.

### Results and discussion

In order to test the performance of the system and the software algorithms, a number of acid and base standard solutions and real samples were determined; the results were compared with those obtained by widely used recommended analytical procedures.

*Titration of strong acids*

The system was applied to the titration of HCl standards (19–250 μeq) by using different hydroxyl generation intensities (5, 10 and 15 mA). The results obtained are shown in table 1. As can be seen, there is good agreement between the expected and found values, as well as good reproducibility. Figure 2 shows an experimental titration curve for one of these experiments, captured directly from the computer screen. The equivalent point (25·07 μeq), indicated by the vertical line, was automatically detected by means of the Savitzky–Golay algorithm. The corrected value, obtained by subtracting 4·18 μeq of the blank, was 20·89 μeq.

The detection limit, calculated as five times the standard deviation of the blank, was 1 μeq.

*Table 1. Results obtained in the coulometric titration of HCl standards (three replicates) at different electrogeneration intensities.*

| | Found | | | | | |
|---|---|---|---|---|---|---|
| | 5 mA | | 10 mA | | 15 mA | |
| Added (μeq) | (μeq) | (% RSD) | (μeq) | (% RSD) | (μeq) | (% RSD) |
| 19·44 | 20·14 | 1·7 | 20·47 | 0·6 | 20·39 | 0·9 |
| 48·60 | 49·39 | 1·7 | 49·00 | 0·4 | 49·36 | 0·8 |
| 97·20 | 99·00 | 0·8 | 98·68 | 0·5 | 99·11 | 0·2 |
| 243·00 | 246·81 | 0·8 | 246·18 | 1·3 | 247·31 | 0·6 |

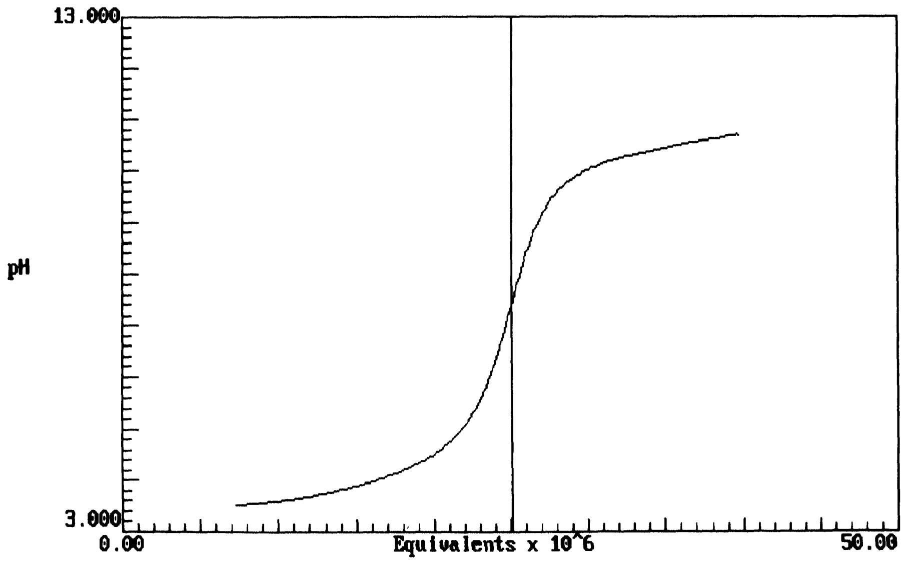

*Figure 2. Titration curve for 2 ml of HCl 0·01 N ($i = 10$ mA, continuous method). End-point of 25·07 μeq is indicated by a vertical line.*

*Table 2. Results obtained in the coulometric titration of various drinking waters (three replicates, $i = 10$ mA).*

| Sample | Reference method (meq/l) | Reference method (% RSD) | Coulometric method (meq/l) | Coulometric method (% RSD) |
|---|---|---|---|---|
| Tap 1† | 5·33 | 0·6 | 5·37 | 0·9 |
| Tap 2† | 2·55 | 0·9 | 2·71 | 0·6 |
| Tap 3† | 5·30 | 0·8 | 5·52 | 0·8 |
| Tap 4† | 5·62 | 0·5 | 5·78 | 0·8 |
| Tap 5† | 5·43 | 1·3 | 5·50 | 1·4 |
| Mineral‡ | 2·31 | 1·7 | 2·34 | 1·0 |
| Tank§ | 2·09 | 1·1 | 2·08 | 0·7 |

† From different districts of Palma de Mallorca.
‡ Binifaldó, Mallorca.
§ María de la Salut, Mallorca.

*Titration of strong bases*

NaOH standards containing 20, 50 and 100 μeq were assayed with electrogenerated protons. The results obtained indicate that such standards can be titrated individually; however, the gradual carbonation of the samples from atmospheric $CO_2$ poses serious problems in analysing large numbers of samples. Overcoming this problem requires taking special precautions to avoid contact of the samples with the air, which results in a more complicated instrumental set-up.

*Determination of water alkalinity*

The system was used for the determination of the overall alkalinity of drinking waters (Palma de Mallorca, Spain). The results were contrasted with those obtained by manual titration of the same samples with 0·1 N HCl using Methyl Orange and phenolphthalein as indicators [8]; see table 2. As can be seen, there is good agreement between the two sets. This type of sample is not affected by the waiting time, and so can be assayed with the autosampler.

*Determination of phosphoric acid in cola drinks*

The coulometric system was also used to determine the phosphoric acid content of two pre-decarbonated cola drinks (two unknown samples submitted for analysis in our laboratory). The results were compared with those obtained by the well-known spectrophotometric molybdenum heteropolyacid blue method [9]. They are listed in table 3, which shows the two sets to be quite consistent. Figure 3 shows an experimental titration curve captured from the computer screen. This indicates the successive titration of the two protons of phosphoric acid. The first equivalent point corresponded to 154 ppm P, whereas the second corresponded to 179 ppm P. The former agrees with the 158 ppm P obtained by the manual colorimetric method.

*Table 3. Results obtained in the analysis for phosphoric acid in cola drinks (unknown samples, submitted for analysis to the authors' laboratory).*

| Sample | Reference method (ppm P) | Coulometric method (ppm P) | Coulometric method (% RSD) |
|---|---|---|---|
| Cola 1 | 120 | 117 | 1·4 |
| Cola 2 | 158 | 159 | 3·0 |

$i = 10$ mA, five replicates.

*Determination of the total acidity of wines*

The acidity of Spanish wine samples (Rioja red wine and Valdepeñas white wine) was determined up to pH 7. The results are compared with those obtained by potentiometric titration with 0·1 N NaOH up to pH 7 [10] in table 4.

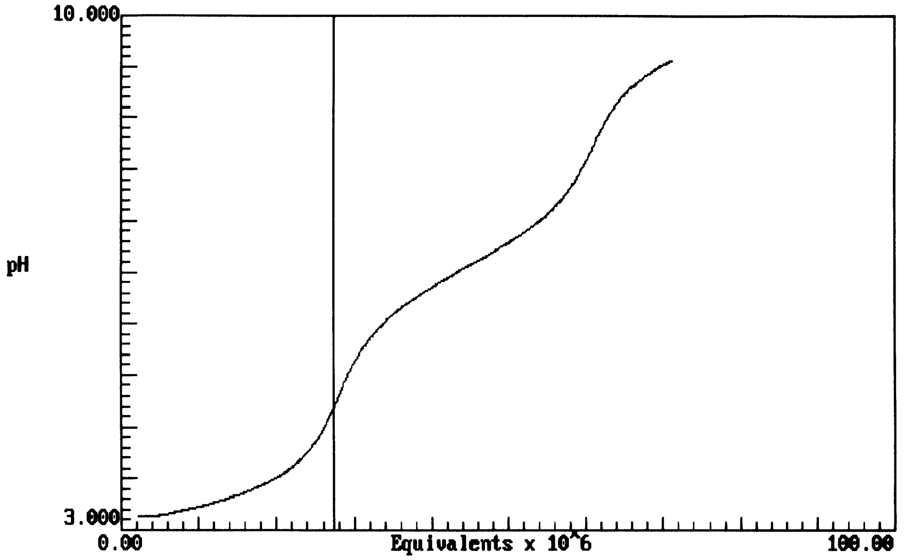

*Figure 3. Titration curve for 5 ml of Cola 2 (i = 10 mA, continuous method). The first end-point of 27·3 μeq is indicated by the vertical line (the program shows a vertical line only for one end-point in each data treatment).*

*Table 4. Results obtained in the determination of wine acidity.*

| Sample | Reference method (meq/l) | (% RSD) | Coulometric method (meq/l) | (% RSD) |
|---|---|---|---|---|
| White wine (Valdepeñas, Spain) | 57·97 | 0·8 | 59·90 | 0·5 |
| Red wine (Rioja, Spain) | 58·37 | 1·8 | 59·36 | 0·7 |

$i$ = 10 mA, five replicates.

### Conclusions

The proposed instrumental set-up and software are suitable for acid–base titrations on a variety of samples. The system has all the advantages of coulometric titrations (for example no need to prepare, standardize or store titrant solutions), and allows the procedure to be implemented in a fully automatic fashion, thereby allowing larger numbers of analyses to be performed without human intervention – up to 13 samples can be assayed in one session at an average rate of 5 min/sample. In addition, the high sensitivity of the determinative method allows analyses to be made on very small amounts of sample.

The proposed system is inexpensive because it is constructed from commercially available parts and features very low maintenance costs.

### Acknowledgements

The authors are indebted to the DGICyT (Spanish Council for Research in Science and Technology) for financial support granted through Project PA 86-0003.

### References

1. Zagidullina, G. Z. and Yunnikova, M. V., *Fermenth. Spirt. Prom-st.*, **5** (1987), 8.
2. Su, X., Chen, H. and Yang, S., *Xiamen Daxue Xuevao, Ziran Kexueban*, **26**(5), (1987), 605.
3. Taylor, P. J. and Brown, D. M., *Comput. Appl. Lab.*, **1**(3), (1983), 179.
4. Pap, T., Hajos, P. and Inczedy, J., *Magyar Kemiai Folyoirat*, **87**(1), (1981), 36.
5. Cladera, A., Caro, A., Estela, J. M. and Cerdà, V., *International Journal of Environmental Analytical Chemistry* (in press).
6. Savitzky, A. and Golay, M. J. E., *Analytical Chemistry*, **36** (1964), 1627.
7. Steiner, J., Termonia, Y. and Deltour, J., *Analytical Chemistry*, **44** (1972), 1906.
8. Golterman, H. L., Methods for chemical analysis of fresh waters. *IBP Handbook No. 8* (Blackwells, Oxford, 1970).
9. Vogel, A., *A Text-book of Quantitative Inorganic Analysis*, (Longman, London, 1961).
10. OIV, Récueil des méthodes internationales d'analyse des vins. **A10**, 1–3 (1969).

